# Challenges in the clinical development of PI3K inhibitors

**DOI:** 10.1111/nyas.12060

**Published:** 2013-03-28

**Authors:** Cristian Massacesi, Emmanuelle Tomaso, Nathalie Fretault, Samit Hirawat

**Affiliations:** 1 Novartis OncologyParis, France; 2 Novartis Institutes for BioMedical Research, Inc.Cambridge, Massachusetts; 3 Novartis Pharmaceuticals CorporationFlorham Park, New Jersey

**Keywords:** PI3K antagonists, PI3K inhibitors, patient selection, research design

## Abstract

The PI3K/Akt/mTOR pathway is one of the most frequently dysregulated signaling pathways in cancer and an important target for drug development. PI3K signaling plays a fundamental role in tumorigenesis, governing cell proliferation, survival, motility, and angiogenesis. Activation of the pathway is frequently observed in a variety of tumor types and can occur through several mechanisms. These mechanisms include (but are not limited to) upregulated signaling via the aberrant activation of receptors upstream of PI3K, amplification or gain-of-function mutations in the *PIK3CA* gene encoding the p110α catalytic subunit of PI3K, and inactivation of *PTEN* through mutation, deletion, or epigenetic silencing. PI3K pathway activation may occur as part of primary tumorigenesis, or as an adaptive response (via molecular alterations or increased phosphorylation of pathway components) that may lead to resistance to anticancer therapies. A range of PI3K inhibitors are being investigated for the treatment of different types of cancer; broad clinical development plans require a flexible yet well-structured approach to clinical trial design.

## Introduction

**Figure 1:**
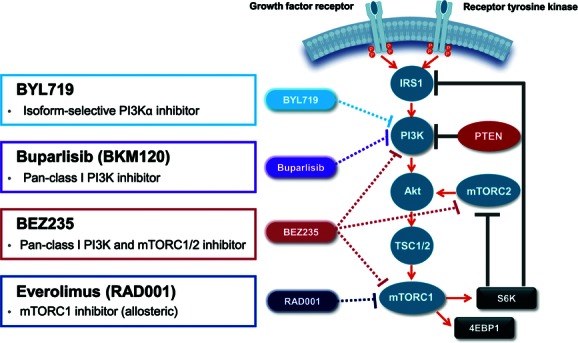
The PI3K/Akt/mTOR pathway and inhibitors that target it. IRS1, insulin receptor substrate 1; mTORC, mammalian target of rapamycin complex; PI3K, phosphatidylinositol 3-kinase; PTEN, phosphatase and tensin homolog; TSC, tuberous sclerosis protein.

The widespread and pivotal role of the PI3K pathway in cancer has inspired the active development of a spectrum of drugs that target various components of the pathway. These drugs include allosteric mTORC1 inhibitors, Akt inhibitors, inhibitors of all four class I PI3K isoforms (so-called pan-class I PI3K inhibitors), dual pan-class I PI3K and mTORC1/2 inhibitors, and, most recently, isoform-specific PI3K inhibitors. Novel compounds in clinical development by Novartis include the pan-PI3K inhibitor buparlisib (BKM120), the dual pan-PI3K/mTORC1/2 inhibitor BEZ235, and the selective p110α inhibitor BYL719. In addition, the mTORC1 inhibitor everolimus is already approved for use in several types of cancer (Fig. 1).

Due to the complexity of the PI3K pathway, and the extensive cross-talk with other pathways, one of the greatest challenges in PI3K inhibitor development involves identifying the patients that will benefit most from treatment. Early-phase single-agent trials with PI3K inhibitors have yet to identify a consistent and distinct association between typical PI3K pathway alterations (*PIK3CA* mutation and PTEN loss) and response to therapy. This may partly be due to the heterogeneous range of cancers treated in these trials. The PI3K pathway interacts with other signaling pathways at several points, and these interactions are known to vary in a tissue-specific manner. Therefore, the capability of predictive biomarkers, and the effectiveness of different types of PI3K inhibitors, may also vary across tumor types. As the development of PI3K inhibitors progresses from mid to late phase and expands into tumor-specific studies, Novartis is employing a flexible approach to biomarker-driven study design, using a range of strategies based on the phase of drug development, the type of PI3K inhibitor, the tumor type under investigation, and the specific context of treatment. This mini-review summarizes four distinct approaches to study design and describes the rationale for their use in terms of the currently enrolling trials with Novartis PI3K inhibitors.

## Patient stratification based on PI3K pathway status (breast cancer)

PI3K inhibitors have demonstrated encouraging preliminary activity in the treatment of metastatic breast cancer, with responses observed in patients with and without *PIK3CA* and *PTEN* alterations.^1^,^2^ Evidence for the activity of PI3K inhibitor–based therapy in breast cancer has been drawn from a phase I study in patients with hormone receptor (HR)–positive metastatic breast cancer.^3^ In this trial, patients received continuous (*n* = 20) or intermittent (five days on, two days off; *n* = 31) doses of buparlisib in combination with letrozole. The majority of patients (*n* = 43) had received prior aromatase-inhibitor therapy. The clinical benefit rate (complete responses plus partial responses plus stable disease) at six months was 30% and 29% in the continuous and intermittent cohorts, respectively. A correlation between duration of response or clinical benefit and the presence of *PIK3CA* mutation has yet to be observed in either cohort.

Given the aforementioned findings, the approach Novartis has taken in breast cancer has been to develop trials that are adequately powered to prospectively investigate efficacy in both the population as a whole and in the subpopulation of patients with PI3K pathway alterations. BELLE-2 (NCT01610284) is a multicenter phase III, placebo-controlled study of buparlisib plus fulvestrant that will enroll 842 postmenopausal women with HR-positive/HER2-negative advanced breast cancer whose disease has progressed on or after aromatase-inhibitor therapy, including ≥ 334 patients with PI3K pathway alterations. Enrollment will be stratified by the presence or absence of PI3K pathway activation, defined as *PIK3CA* mutation and/or *PTEN* alteration. BELLE-2 is designed to investigate progression-free survival (PFS) in the population as a whole and/or in the PI3K pathway-activated subpopulation using a gate-keeping procedure based on a graphical approach to address the multiplicity of hypotheses.^4^ The results of this study could provide prospective evidence regarding the use of these biomarkers in predicting response to PI3K inhibitor therapy. Other trials with buparlisib in breast cancer are employing similar approaches, including a placebo-controlled phase II trial with paclitaxel in the first-line treatment of HER2-negative metastatic breast cancer (BELLE-4; NCT01572727), and a phase II trial of neoadjuvant paclitaxel plus trastuzumab, with and without buparlisib (Neo-PHOEBE) in HER2-overexpressing breast cancer patients.

## Nonselective enrollment and mandatory tissue collection (prostate cancer and glioblastoma)

Another strategy is to conduct early-phase trials in tumor types with high frequencies of PI3K pathway alterations and strong preclinical evidence supporting the potential efficacy of PI3K-inhibition treatment. These trials enroll patients regardless of PI3K pathway status; however, enrollment is dependent upon the mandatory provision of tumor tissue, which can be used for exploratory *post hoc* analyses. Castration-resistant prostate cancer (CRPC) is one such tumor type being investigated using this strategy.

PTEN loss is one of the most frequent molecular aberrations to occur in prostate cancer, and ∼70% of metastatic cases have some form of alteration in the PI3K pathway. This high frequency of alterations supports the rationale for investigating PI3K inhibitors in this tumor type. Furthermore, interaction and reciprocal feedback regulation between the androgen receptor and PI3K pathways has been suggested as a potential mechanism of resistance to androgen-deprivation therapy in CRPC. PI3K inhibitors may therefore have the potential to reverse resistance in this context. In preclinical experiments, the combination of BEZ235 and enzalutamide (an androgen-receptor antagonist) demonstrated near-complete tumor regression in a PTEN-deficient murine model and in human prostate cancer xenografts.^5^ A phase Ib proof-of-concept trial of BEZ235 or buparlisib in combination with abiraterone acetate is currently enrolling patients with CRPC after progression on abiraterone acetate (NCT01634061).

Glioblastoma multiforme (GBM) is another tumor type with a high frequency of PI3K pathway alterations, with PTEN loss reported in up to 35% of cases. Buparlisib has demonstrated an ability to cross the blood–brain barrier and inhibit the PI3K pathway in the brain, and has shown synergy with temozolomide and docetaxel in murine xenografts of *PTEN*-null GBM.^6^ A phase I trial is investigating buparlisib in combination with adjuvant temozolomide and with concomitant radiotherapy and temozolomide in newly diagnosed GBM (NCT01473901). Two other ongoing phase I/II trials are investigating single-agent buparlisib or the combination of buparlisib and bevacizumab in patients with relapsed disease (NCT01339052 and NCT01349660, respectively). Enrollment in both of these trials is dependent on the provision of tumor biopsy material for the analysis of PI3K pathway alterations.

## Preselection of patients with PI3K pathway activation—enrichment strategy (nonsmall cell lung cancer)

Certain contexts may necessitate the design of trials that selectively recruit patients with PI3K pathway alterations only. Lung cancer treatment has recently moved toward a customized approach based on the molecular characteristics of tumors: patients with *EGFR* mutations may show improved benefit from EGFR tyrosine kinase inhibitors (TKIs; e.g., erlotinib and gefitinib), and those with *ALK* translocations from ALK inhibitors (e.g., crizotinib). Preclinical experiments have suggested that PI3K pathway alterations may predict a differential response to PI3K inhibitors in models of nonsmall cell lung cancer (NSCLC),^7^ and PI3K pathway activation has been identified as one of the factors driving resistance to EGFR TKIs in preclinical models.^8^ An ongoing phase II study (NCT01297491) is therefore evaluating single-agent buparlisib versus docetaxel or pemetrexed in patients with squamous or nonsquamous metastatic NSCLC with PI3K pathway alterations (*PIK3CA* mutation and/or *PTEN* alteration). Patients who have been pretreated with one or two prior antineoplastic treatments are eligible.

Isoform-specific PI3K inhibitors may theoretically offer an improved therapeutic window and narrower toxicity profile compared with pan-PI3K inhibitors. The selective PI3Kα inhibitor BYL719 has shown preferential sensitivity in *PIK3CA*-mutated cell lines, and a first-in-man study with this agent (NCT01387321) is enrolling patients with *PIK3CA* mutation or amplification only to maximize the potential benefit of treatment.^9^ Preliminary results from this phase I trial of single-agent BYL719 in patients with advanced solid tumors suggests a favorable safety profile, with two confirmed partial responses observed (one each in patients with HR-positive breast cancer and cervical cancer).^9^

## Enrollment of patients that have progressed on mTORC1 inhibitor-based therapy

The BOLERO-2 trial showed substantial improvements in PFS with the combination of everolimus and exemestane, compared with exemestane alone, in patients with advanced HR-positive breast cancer who had progressed on nonsteroidal aromatase inhibitors.^11^ Despite these improvements in PFS, resistance to the combination of everolimus and exemestane can occur. Inhibition of mTORC1, but not mTORC2, can cause paradoxical reactivation of the PI3K pathway through the alleviation of feedback loops dependent on S6K.^10^ PI3K inhibitors, which target the pathway upstream of mTORC1, may therefore show utility in contexts in which mTORC1 inhibitors are unsuccessful or no longer effective. The potential use of PI3K inhibitors in the post-mTORC1 inhibitor treatment setting is being investigated in BELLE-3 (NCT01633060), a placebo-controlled phase III study to investigate the safety and efficacy of buparlisib plus fulvestrant in postmenopausal women with HR-positive/HER2-negative advanced breast cancer who have received aromatase-inhibitor treatment and progressed on or after mTORC1 inhibitor-based therapy. Like BELLE-2, BELLE-3 is stratifying enrolling patients according to PI3K pathway activation status, to investigate the treatment effect in patients with PI3K pathway activation and/or the population as a whole.

## Summary

The burgeoning field of PI3K inhibitor development is associated with many ongoing challenges. PI3K signaling is complex and can be modulated by crosstalk with other kinase cascades, such as the Ras/Raf/MEK pathway. This complexity is further compounded by tissue-specific effects, which may complicate the identification of predictive biomarkers.

It remains unclear whether preclinical observations of improved responses to PI3K inhibitors in tumors with *PIK3CA* and *PTEN* alterations will be borne out in clinical trials. Early-phase, single-agent trials with PI3K inhibitors have yet to establish a consistent and distinct association between the most common alterations in the PI3K pathway and response to therapy. Explanations for this are numerous and include heterogeneity in the patient population, use of archival specimens for biomarker assessment, and a low number of responses to single-agent PI3K inhibitors. Future trials of PI3K inhibitors as combination therapy in more homogenous patient populations may be more likely to establish a link between typical PI3K alterations and clinical response.

The successful evaluation of PI3K pathway biomarkers is complicated by many factors, such as observations of discordance between primary and metastatic lesions and issues with intratumoral heterogeneity in molecular alterations. It is possible that future studies will require the prospective collection of biopsies immediately before and after study treatment to address these difficulties. Advances in noninvasive technologies, such as circulating DNA and/or tumor cell analysis, may eventually allow this approach. Future studies may also benefit from deeper analyses into pathway alterations and signaling, such as those offered by high-throughput, next-generation sequencing and phosphoproteomic analyses.

Finally, the spectrum of different PI3K inhibitors also presents its own dilemma: How are the optimal indications for each class of inhibitor identified? Whereas more selective inhibitors may offer improved therapeutic windows and narrower toxicity profiles, certain tumor types or treatment contexts may require more comprehensive inhibition of the PI3K pathway. These assessments will include the identification of the optimal dose and dosing schedule of each inhibitor and the tumor types in which they can best be used, and will be planned based on robust preclinical evidence.

In conclusion, PI3K inhibitors show great promise in the treatment of a wide range of cancers; a well-structured approach to study design will be required to maximize the potential of this exciting class of therapy.
